# Reducing X-Ray Induced Oxidative Damages in Fibroblasts with Graphene Oxide

**DOI:** 10.3390/nano4020522

**Published:** 2014-06-24

**Authors:** Yong Qiao, Peipei Zhang, Chaoming Wang, Liyuan Ma, Ming Su

**Affiliations:** Department of Biomedical Engineering, Worcester Polytechnic Institute, Worcester, MA 01609, USA; E-Mails: qiaoyong83@gmail.com (Y.Q.); pzhang2@wpi.edu (P.Z.); cwang4@wpi.edu (C.W.)

**Keywords:** X-ray, graphene oxide, DNA damage, oxidative, reactive oxygen species (ROS)

## Abstract

A major issue of X-ray radiation therapy is that normal cells can be damaged, limiting the amount of X-rays that can be safely delivered to a tumor. This paper describes a new method based on graphene oxide (GO) to protect normal cells from oxidative damage by removing free radicals generated by X-ray radiation using grapheme oxide (GO). A variety of techniques such as cytotoxicity, genotoxicity, oxidative assay, apoptosis, γ-H2AX expression, and micro-nucleus assay have been used to assess the protective effect of GO in cultured fibroblast cells. It is found that although GO at higher concentration (100 and 500 µg/mL) can cause cell death and DNA damage, it can effectively remove oxygen free radicals at a lower concentration of 10 µg/mL. The level of DNA damage and cell death is reduced by 48%, and 39%, respectively. Thus, low concentration GO can be used as an effective radio-protective agent in occupational and therapeutic settings.

## 1. Introduction

Radiation therapy, using external X-ray beams, relies on free radicals, generated from water radiolysis, to damage DNA [1]. Tumor killing selectivity is due to reduced ability of cancer cells to repair damaged DNA [2,3,4,5,6,7,8]. A challenge of external beam X-ray radiation therapy is the high radiation doses needed to kill tumor cells also damages surrounding healthy tissues. Although many beam techniques have been used to enhance radio-sensitivity of tumor, and minimize X-ray doses on normal cells, damages to normal cells already occur at time when radiation dose is sufficient to kill tumor due to similar amounts of water contents in normal cells and in cancerous cells [9]. Reactive oxygen species (ROS) can damage many cellular components, including proteins, lipids and DNA [10,11]. Oxidative damage to plasma membrane lipids can stimulate cytoplasmic signal pathways to activate apoptosis [12]. Free radicals also extract hydrogen atoms from DNA, causing a wide range of DNA damage such as strand breaks, base alteration, and DNA cross-links [13]. Radio-protective chemicals can be used as free radical scavengers to protect normal cells from damage, allowing higher radiation doses to be used [14,15,16,17,18]. As DNA damage can lead to histone (γ-H2AX) over-expression, cell apoptosis, and micronucleus, a series of techniques can be used to characterize X-ray induced damages to cells.

Grapheme oxide (GO) is a water-solvable, non-toxic, and biodegradable material [19]. It has been used to deliver anticancer drugs to cancer cells for chemotherapeutic purpose [20,21,22,23]. GO nanoparticles can be modified to bind with certain tissue [24,25]. All carbon atoms in GO are exposed, and those at the edge have a higher reactivity than those in the plane. GO’s open format allows for efficient capture of oxygen free radicals with carbon atoms on the edge. This generates carbon dioxide, which can be dissolved in body fluids [26,27]. Therefore, GO can be used as a new type of free radical scavenger. This paper describes the use of GO in removing reactive oxygen species (ROS) generated upon X-ray radiation. Normal human fibroblast cells are used as a model system. A battery of techniques has been employed to quantify radiation induced changes at molecular (DNA and protein) and cellular levels.

## 2. Results and Discussion

**Figure 1:**
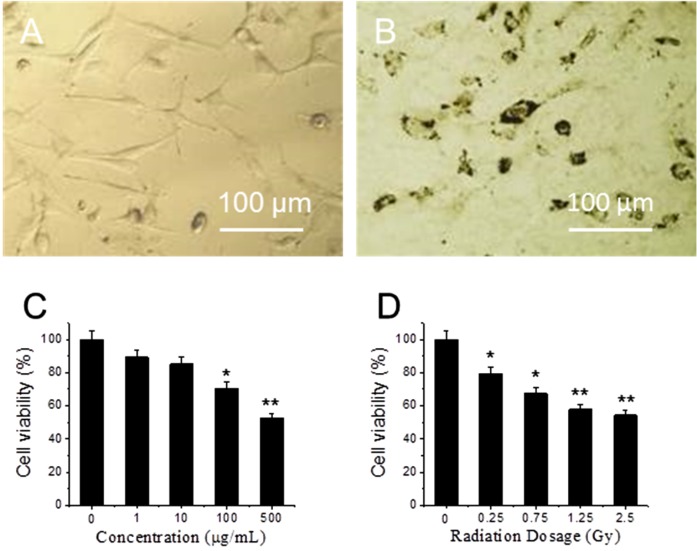
Optical images of cells (**A**) and cells treated with graphene oxide (GO) for 24 h (**B**); cytotoxicity of cells treated with different concentrations of GO (**C**); and exposed to different dose of X-ray (**D**). “*” (*p* < 0.05) and “**” (*p* < 0.01) represent significant difference and extra significant difference, respectively.

Figure 1A,B shows optical images of fibroblasts cultures that were treated with 0 and 10 μg/mL GO for 24 h at 37 °C. Compared to 1A, the morphology of cells in 1B does not change, though many GO particles are attached on the cell surface. After replacing GO-containing medium, cells are rinsed by PBS for three times and exposed to X-ray radiation. MTT assay results show that viability of GO-treated cells decrease as GO concentration increases from 1 to 500 μg/mL (Figure 1C). At low concentration (1 and 10 μg/mL), GO does not have significant toxicity [28,29]. At high concentrations, 100 μg/mL and 500 µg/mL, cell viability decreases, where 30% and 48% of cells are killed respectively. In order to test cytotoxicity induced by X-ray radiation, fibroblasts are exposed to different doses of X-ray radiation. Figure 1D shows cell viability decreases as X-ray dose increases from 0 to 2.5 Gy. When the dose is in the range from 0 to 0.75 Gy, cell viability shows significant difference decreases (*p* < 0.05); while when the dose is higher than 1.25 Gy, cell viability shows extra significant difference decreases (*p* < 0.01). As low dose and low GO concentration can decrease side effects, 10 μg/mL GO and 1.25 Gy X-ray have been chosen in the following experiment.

**Figure 2:**
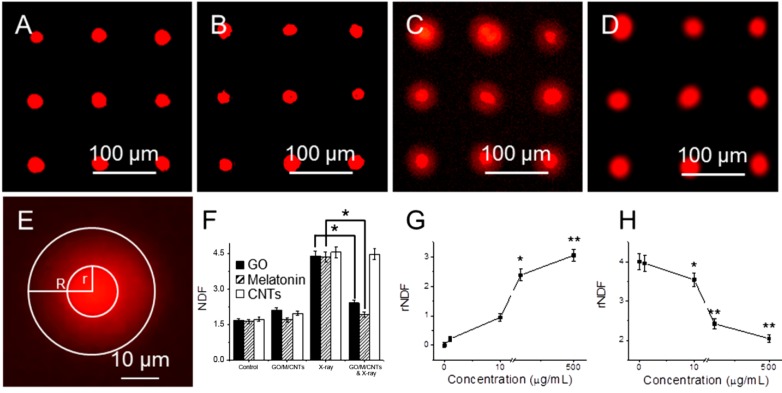
Genotoxicity of cells treated with GO and X-ray irradiation with halo assay. Fluorescent images of arrayed cells (**A**); cells treated with 10 µg/mL GO (**B**); cells exposed to 1.25 Gy X-ray (**C**); and cells treated with GO and then exposed to 1.25 Gy X-ray (**D**); an enlarged image shows that halo and nucleus (**E**); the NDF values of cells after different treatment (**F**); the rNDF values of cells treated with different concentration of GO without (**G**) and with 1.25 Gy X-ray radiations (**H**). “*” (*p* < 0.05) and “**” (*p* < 0.01) represent significant difference and extra significant difference, respectively.

DNA damage in cells has been studied with HaloChip assay after embedding cells in agarose gel [30]. SYBR Green I dye is used to label DNA. Figure 2A shows a fluorescent image of the control group, which is not treated with GO, and not exposed to X-ray. Here there is no DNA diffusion from nucleus. Figure 2B shows a fluorescent image of cell array after incubated with 10 µg/mL GO for 24 h, where GO does not cause DNA damage. Figure 2C shows fluorescent image of arrayed cells that are exposed to 1.25 Gy X-ray radiations, where more DNA damage can be found to form diffusive halo around nuclei. Figure 2D shows fluorescent image of arrayed cells that are pretreated with GO and then exposed to X-ray radiation. A relative nuclear diffusion factor (rNDF) is used to quantify the level of DNA damage. rNDF is defined as rNDF = (*R*^2^ − *r*^2^)/*r*^2^, where R and r are the radii of large circle and small circle in Figure 2E, respectively. Figure 2F shows rNDF values of (1) cells; (2) cells treated with 10 µg/mL GO; (3) cells exposed to 1.25 Gy X-ray; and (4) cells treated with 10 µg/mL GO, and then exposed to 1.25 Gy X-ray. The level of DNA damage in sample (4) is lower than that in sample (3), suggesting that GO treatment can effectively prevent X-ray induced DNA damage. In addition, cells were also treated with 10 µg/mL of melatonin and carbon nanotubes (CNTs), and exposed to 1.25 Gy X-ray. The NDF value of melatonin is slightly smaller than GO, suggesting that GO is slightly weaker than melatonin in removing free radicals. This is likely due to the fact that melatonin (being molecules) can be dispersed better than GO (macromolecules). Meanwhile, the NDF value of sample exposed to CNTs and X-ray is similar as that treated with X-ray alone. So CNTs cannot remove ROS generated by X-ray as effective as GO due to its close structure. Figure 2G shows rNDF values of cells treated with different concentration of GO for 24 h, where concentration dependent DNA damage can be found. At low concentrations (1 and 10 μg/mL), GO itself does not cause DNA damage; but at high concentration (100 and 500 μg/mL), GO can cause significant DNA damage. Figure 2H shows rNDF values of cells treated with different concentration of GO for 24 h, and then exposed to 1.25 Gy X-ray, where DNA damage can be reduced at all concentration of GO, with less DNA damage at high GO concentration.

**Figure 3:**
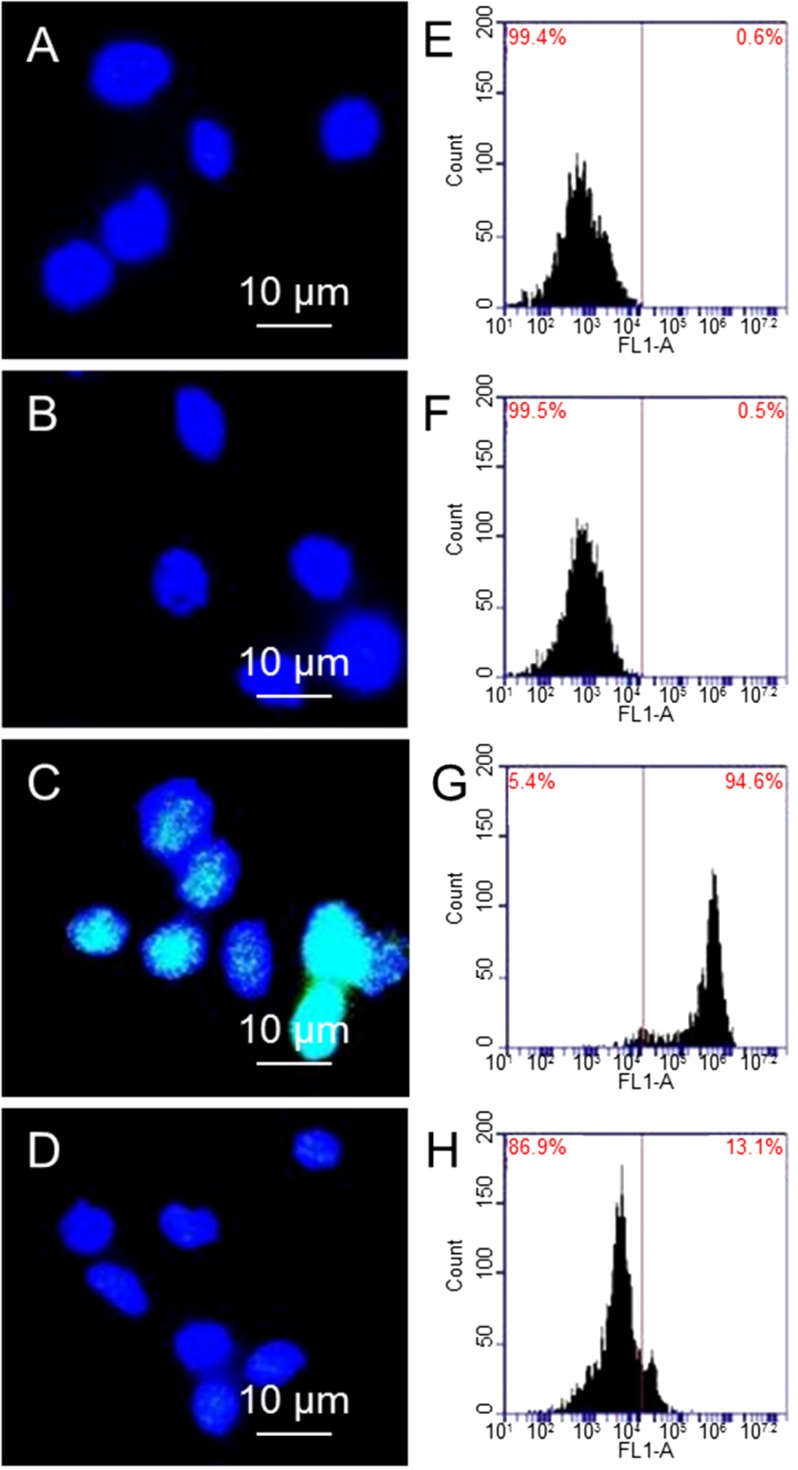
Immunostaining images of cells (**A**); cells treated with GO (**B**); cells exposed to X-ray (**C**); and cells treated with GO and then exposed to X-ray (**D**); flow cytometry results of cells after different treatments (**E**–**H**).

Once damaged, a group of DNA repair proteins accumulate around damaged sites. γ-H2AX is a protein that is related to repairing DNA double strand breaks [31,32]. Detecting γ-H2AX is one way to quantify double strand breaks of DNA. Four samples are studied: (1) cells; (2) cells treated with 10 µg/mL GO; (3) cells exposed to 1.25 Gy X-ray and (4) cells treated with 10 µg/mL GO and then exposed to 1.25 Gy X-ray. DNAs are stained with DAPI for observation of nuclei. Figure 3 shows the immunostaining and flow cytometry results of γ-H2AX expression, where γ-H2AX is shown in green and DNA is shown in blue. Figure 3A,B are fluorescence images of cells and cells treated with 10 µg/mL GO alone, where DNA (blue color) can be seen clearly; while γ-H2AX (green color) cannot be seen due to small amount of γ-H2AX. In comparison, cells treated with 1.25 Gy X-ray has shown strong green fluorescence (3C). There is weak green fluorescence from cells that are treated with 10 µg/mL GO before exposing to 1.25 Gy X-ray (3D). Figure 3E–H shows the flow cytometry results, where the shift of the peak towards the right is proportional to the level of γ-H2AX. Comparing cells alone (3E) and cells treated with GO (3F), cells exposed to X-ray (3G) show a significant shift towards the right, suggesting a large population of cells have high levels of γ-H2AX, as well as double DNS strand breaks. X-ray radiation of GO treated cells does not shift the peak much compared to cells alone, or cells treated with 10 µg/mL GO (3H). Taking cells without GO treatment and without X-ray exposure as standard (0.6% γ-H2AX), cells treated with GO show 0.5% γ-H2AX, cells exposed to X-ray show 94.6% γ-H2AX, and cells treated with GO and then exposed to X-ray show 13.1% γ-H2AX. These results confirm GO can significantly reduce X-ray induced DNA double strand breaks.

**Figure 4:**
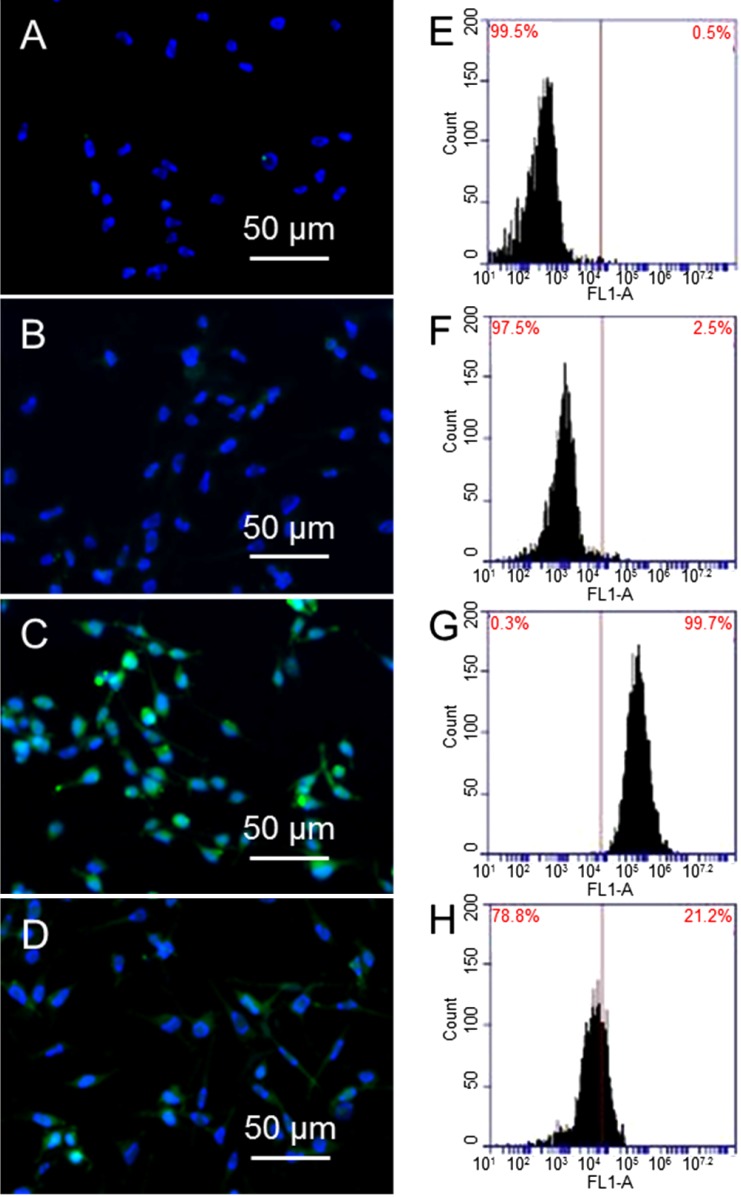
Oxidative stress induced by GO and X-ray. Fluorescent images of cells (**A**); cells treated with 10 µg/mL GO (**B**); cells exposed to 1.25 Gy X-ray (**C**); and cells treated with 10 µg/mL GO and then exposed to 1.25 Gy X-ray (**D**); Flow cytometry results of cells after different treatment: cells (**E**); cells treated with GO (**F**); cells exposed to 1.25 Gy X-ray (**G**); and cells treated with GO and exposed to X-ray (**H**).

X-ray radiation can generate ROS [33], which can cause alteration of membrane lipids, proteins, and nucleic acids. The effect of GO in reducing ROS generation is tested as follows. Fibroblasts are treated with 10 µg/mL GO and exposed to 1.25 Gy X-ray radiation, followed by H2DCFDA staining and flow cytometry. In addition, DNA is stained with Hoechst 33342 (Life Technologies, Eugene, OR, USA) for observation of nucleus. Figure 4A,B are fluorescence images of untreated cells and cells treated with 10 µg/mL GO alone, where DNA in nucleus (blue color) can be seen clearly; while carboxy-DCF (green color) cannot be seen due to small amount of ROS. In comparison, cells treated with 1.25 Gy X-ray show strong green fluorescence (Figure 4C). Most importantly, there is weak green fluorescence from cells that are treated with 10 µg/mL GO before exposing to 1.25 Gy X-ray (Figure 4D). Flow cytometry results are shown in Figure 4E–H, where the peaks shift toward the right is proportional to the level of ROS. Compared to untreated cells (4E) and cells treated with GO (4F), cells exposed to X-ray (4G) show a significant shift towards right, suggesting a large population cells have high level of ROS. X-ray irradiation of GO treated cells does not shift the peak much compared to cells alone, or cells treated with 10 µg/mL GO (4H). Taking cells without GO treatment and without X-ray exposure as standard (0.5% ROS), cells treated with GO show 2.5% ROS; cells exposed to X-ray show 99.7% ROS, and cells treated with GO and then exposed to X-ray show 21.2% ROS. These results confirm that GO can significantly reduce X-ray induced ROS generation.

**Figure 5:**
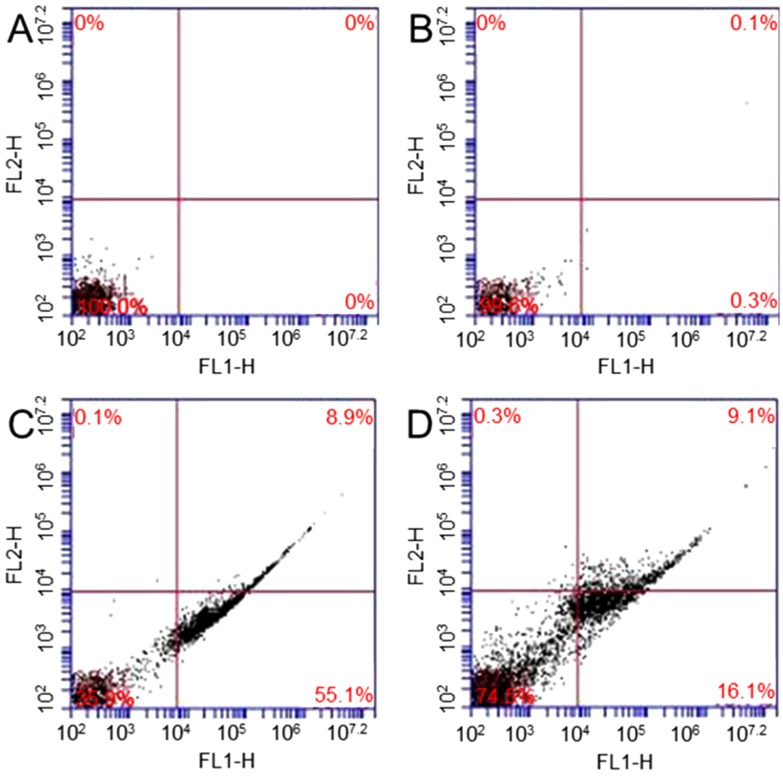
Flow cytometry evaluation of cell apoptosis: cells (**A**); cells treated with GO (**B**); cells exposed to 1.25 Gy X-ray (**C**); and cells treated with 10 µg/mL GO and then exposed to 1.25 Gy X-ray (**D**).

Annexin V-FITC apoptosis detection kit is used to study X-ray irradiation, GO induced apoptosis, and necrosis of fibroblasts [34]. Four samples are studied: (1) cells; (2) cells treated with 10 µg/mL; (3) cells exposed to 1.25 Gy X-ray and (4) cells treated with 10 µg/mL GO and then exposed to 1.25 Gy X-ray. The percentages of apoptosis are 0% and 0.3% in sample 1 (Figure 5A) and sample 2 (Figure 5B), respectively. Figure 5C shows that 55.1% of cells exposed to X-ray undergo apoptosis. Figure 5D shows that 16.1% of cells treated with GO and then exposed to X-ray undergo apoptosis. The results show that no significant change in apoptosis is observed in GO treated cells. After GO treatment, X-ray radiation can only causes a small amount of cells undergo apoptosis.

**Figure 6:**
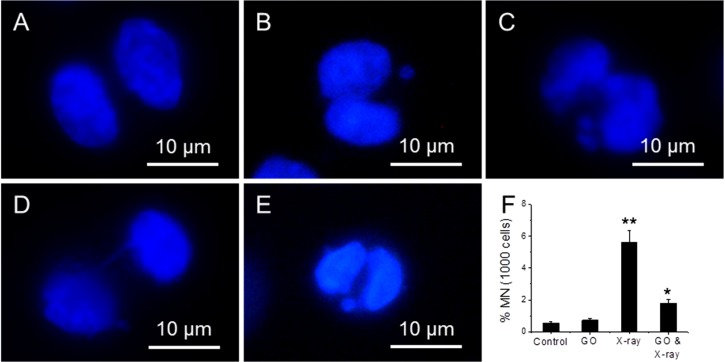
Fluorescence images of cells exposed to X-ray, where a bi-nucleated fibroblast has no micronucleus (**A**); one micronucleus (**B**); two micronuclei (**C**); a nucleoplasmic bridge (NPB) (**D**); and a nuclear bud (NBUD) (**E**); the appearance frequency of micronucleus of four samples (**F**), where cells are arrested at inter-phase stage. “*” (*p* < 0.05) and “**” (*p* < 0.01) represent significant difference and extra significant difference, respectively.

Micronucleus assay can detect chromosome integrity of cells [35]. Four samples have been studied: (1) cells; (2) cells treated with 10 µg/mL; (3) cells exposed to 1.25 Gy X-ray and (4) cells treated with 10 µg/mL GO and then exposed to 1.25 Gy X-ray. Figure 6A–E shows the fluorescence images of cells exposed to X-ray, where a bi-nucleated fibroblast has no micronucleus (A); one micronucleus (B); two micronuclei (C); a nucleoplasmic bridge (NPB) (D); and a nuclear bud (NBUD) (E). Figure 6F shows the appearance frequency of micronucleus for four types of samples, where Cyt B is added to arrest cells at inter-phase stage. 1.25 Gy X-ray radiation can induce a significant increase (5.6%) in micronucleus frequency over untreated cells (0.5%), and 10 µg/mL GO treated cells (0.7%). In comparison, the frequency of micronucleus in GO treated and X-ray exposed cells is 1.8%, which is significantly lower than cells treated with X-ray alone.

## 3. Experimental Section

The following test kits were from Invitrogen (Carlsbad, CA, USA): live/dead assay kit, ROS detection kit, MTT cell proliferation assay kit, DAPI and SYBR green I. The following test kits were from Sigma (St. Louis, MO, USA): Annexin V-FITC apoptosis detection kit, anti-phospho-Histone H2AX antibody, and anti-Rabbit IgG-FITC antibody. Melatonin was from EMD Millipore (Billerica, MA, USA). Trypan blue and phosphate buffer saline were from VWR (West Chester, PA, USA). Low melting point agarose was from Invitrogen. GelBond file was from Lonza (Rockland, ME, USA). RPMI-1640 medium, penicillin/streptomycin, trypsin/EDTA solution, and fetal bovine serum were from Thermo Scientific (Logan, UT, USA). Sulfuric acid, ammonium hydroxide, sodium hydroxide, hydrogen peroxide, NaCl, and absolute ethanol were from Sigma-Aldrich (St. Louis, MO, USA). Cytochalasin B (Cyt B) is from Acros Organics (Morris Plains, NJ, USA). GO is provided by Dr. Hua Zhang at Nanyang Technological University, Singapore. CNTs are from Strem Chemicals (Newburyport, MA, USA).

GO was dispersed in ultrapure water at a concentration of 2 mg/mL and diluted to different concentrations with culture medium prior to cell exposure. An atomic force microscopy (AFM) image confirmed that GO is well-dispersed in water. Fibroblast cells were obtained from American Type Culture Collection (ATCC, Manassas, VA, USA) and cultured in standard conditions (5% CO_2_ in air at 37 °C) in RPMI-1640 medium supplemented with 10% (*v*/*v*) fetal bovine serum and 1% (*v*/*v*) penicillin/streptomycin. After the cell monolayer reached 70%–80% confluence, cells were trypsinized with 0.25% trypsin/0.53 mM EDTA solution at 37 °C for 3 min, followed by adding fresh medium at room temperature to neutralize trypsin. After centrifugation and re-suspension in fresh medium, cell viability was determined by staining with Trypan blue, and cell number was counted with hemocytometer (Horsham, PA, USA). A Mini-X X-ray tube from Amptek (Bedford, MA, USA) with a silver anode operated at 40 kV and 100 µA was used to produce X-rays. A brass collimator was used (2 mm diameter pinhole) to focus X-ray onto the target.

Fibroblast cells were seeded in a 96-microwell plate. After cells attached to the surface, GO solution of different concentrations (0, 1, 10, 100 and 500 µg/mL), was added into each well. After incubation for 24 h, each solution was replaced with fresh medium and exposed to different doses (0, 0.25, 0.75, 1.25 and 2.5 Gy) of X-ray radiation. Then cells were put back in the incubator for overnight prior to MTT assay, which was carried out as follows. The medium in each well was removed and replaced with 100 µL of culture medium. Then 10 µL of 12 mM MTT stock solution was added into each well, and into a negative control (100 µL of medium without treatment). After incubation at 37 °C for 4 h, 100 µL of SDS-HCl solution was added into each well and mixed thoroughly using pipette. After incubation at 37 °C for 6 h, each sample was mixed with pipette, and optical absorbance at 570 nm was recorded.

HaloChip assay was performed as follows. An array of micro-patterns, with desired size, was made on a silicon substrate using the same method described before. Cells were seeded on the substrate at a density of 1 × 10^6^ mL^−1^ and incubated for 30 min. After incubation, unattached cells were rinsed away with phosphate buffered saline (PBS). The substrate with cells was placed on a piece of gelbond and 1.5 mL 1% low melting point (LMT)-agarose was dropped onto the substrate. It was kept at room temperature for 10 min to allow gel solidification. DNA damage was evaluated by single cell halo assay in alkaline condition. After exposure to X-rays, cells were immersed in 0.3 M NaOH for 30 min and stained by diluted ×10,000 SYRB green I solution for 15 min. The stained slide was incubated in deionized water for 5 min to remove unbound dye and imaged with an Olympus IX81 fluorescent microscope. Images were analyzed, using ImageJ (NIH, Bethesda, MD, USA), to derive the relative nuclear diffusion factor (rNDF). Each data set was averaged from at least 50 individual cells.

Reactive oxygen species (ROS) were detected using oxidant-sensitive dye DCFH-DA. After exposure to X-rays, a sufficient amount of 25 μM carboxy-H_2_DCFDA solution was applied to cells adhering to the substrate. Cells were incubated with dye for 30 min at 37 °C, gently washed with PBS three times, and stained with Hoechst 33342 (Life Technologies, Eugene, OR, USA) for 10 min at room temperature. In order to carry out flow cytometry experiments, the treated fibroblasts were trypsinized, washed, and re-suspended in PBS, pH 7.3 at a concentration of ~1 × 10^6^ mL^−1^. The cells were then stained with ROS kit. The stained suspension was shielded from light and stored on ice until analysis. Flow cytometry measurements were performed on samples with a BD Accuri C6 System (BD, San Jose, CA, USA), using a 488-nm laser measuring forward and orthogonal light scatter and green fluorescence. Fluorescence data was obtained from 10,000 viable cells per sample.

Apoptosis was determined by staining cells with Annexin V-FITC apoptosis kit. Briefly, after treatment, with GO and X-ray radiation, fibroblasts were collected, washed twice with cold PBS buffer, and re-suspended in binding buffer (10^6^ mL^−1^). An amount of 5 µL of annexin V-FITC and 10 µL propidium iodide solutions were added to these cells. Cells were incubated at room temperature for 10 min and protected from light. At least 10,000 cells were counted and analyzed using flow cytometry.

Fibroblasts plated in petri dish were used to study subcellular localization and expression of γ-H2AX protein. After GO treatment and X-ray exposure, cells were fixed for 20 min with 4% paraformaldehyde in PBS (pH 7.4) and washed three times with PBS on a shaker. Cells were incubated for 10 min with 0.1% Triton X-100 in PBS, washed with fresh PBS, incubated in blocking buffer (3% bovine serum albumin in PBS) for 1 h at room temperature, and then incubated again for 2 h with primary antibodies against γ-H2AX. After being rinsed by PBS for three times, cells were incubated for an additional 1 h with FITC-conjugated secondary antibody (anti-rabbit IgG-FITC antibody produced in goat). After washing with PBS for three times, cells were stained with 0.2 µg/mL DAPI for 15 min in the dark, washed with PBS for three times, and imaged with fluorescence microscope. Treated fibroblasts were trypsinized, washed, and re-suspended in PBS for flow cytometry experiments. Cells were then incubated together with the antibody. Fluorescence data were obtained from 10,000 viable cells per sample.

Micronucleus assay was performed as follows. After cells were exposed to X-ray, Cyt B was added in a final concentration of 5 µg/mL in medium. After maintaining for 24 h, medium was removed and the treated cells were rinsed with PBS three times. Then cells were fixed with 4% paraformaldehyde in PBS, for 15 min, and washed three times with fresh PBS. After incubation for 10 min, with 0.1% Triton X-100 in PBS and washed, cells are air dried, and stained with 0.2 µg/mL of DAPI for 15 min in the dark. After DAPI staining, cells were washed with PBS for three times, and imaged with fluorescence microscope. A total of 1000 cells were scored at 24 h.

Data sets were treated using OriginPro 8.5 (OriginPro 8.5.0 SR1 b161, OriginLab Corporation, Northampton, MA, USA, 2010) and presented as the mean with standard deviation. The statistical significance of results was determined by an analysis of variance using the SPSS software (SPSS 19.0, IBM, Armonk, NY, USA, 2010). Comparisons between control group and treatment group were based on a *t*-test. Results were considered statistically significant when *p* ≤ 0.05. The results represent the mean of at least three independent experiments. For single cell halo assay, each data point was averaged from at least 50 cells. The mean and error values were calculated using OriginPro 8.5 and all data sets were presented as the mean with standard deviation. The statistical significance of results was determined an analysis of variance using the SPSS software (SPSS 19.0, IBM, Armonk, NY, USA). Comparisons between control group and treatment group were based on a *t*-test.

## 4. Conclusions

A variety of techniques have been used to characterize DNA damage, repair protein expression, reactive oxygen species (ROS), and micronucleus and apoptosis of cells upon X-ray radiation in the presence and in the absence of GO. The results confirm that GO can effectively reduce X-ray radiation induced damage to fibroblast cells and DNA by removing reactive oxygen species (ROS). It is found that GO at high concentration (100 and 500 μg/mL) causes cell death and DNA damage, but can effectively remove ROS at concentration of 10 μg/mL. The level of DNA damage and cell death is reduced by 48%, and 39%, respectively. Thus, low concentrations of GO can be used as an effective radio-protective agent in occupational and therapeutic settings.
